# Honor to Bichat

**Published:** 1846-03

**Authors:** 


*Honor to Bichat—His head mistaken for the head of an Idiot.*—According to a late number of the New York Courier des Etats Unis, says Dr. Tabor, the remains of this celebrated physiologist and medical writer, after having reposed for forty-three years in the old St. Catharine cemetery, have been transposed, with great pomp, to *Pere la Chaise.* On exhuming the remains, the skeleton was found without the head. The grave diggers thought they had mistaken the bones of some decapitated malefactor for those of the celebrated Professor! but this circumstance, on the contrary, did but establish the authenticity of the skeleton. The following was revealed. When Bichat died, his loss caused Professor Roux, his intimate friend and companion in labor, very severe grief. Wishing to have constantly by him, some souvenir of his friend, M. Roux cut off his head. We will say, *en passant,* that this head was some years after presented to the Phrenological Society, which pronounced it that of an idiot! The famous Spurzheim pronounced the same opinion upon Laplace's head. However, a deputation having besought M. Roux to profit the occasion in relinquishing the head of Bichat, he at first refused, but finally consented, and the body was re-interred entire. Furthermore, M. Roux is not the only one who has conceived the idea of thus preserving a material relic of a dear friend. When the famous Broussais died, his son cut off the head, and he keeps it in his study.—*Boston Medical and Surgical Journal.*

